# Biomarkers of Cardiovascular Disease

**DOI:** 10.1155/2017/8208609

**Published:** 2017-11-07

**Authors:** Ying Huang, Kailash Gulshan, Truc Nguyen, Yuping Wu

**Affiliations:** ^1^Department of Cellular and Molecular Medicine, Lerner Research Institute, Cleveland Clinic, 9500 Euclid Ave., Cleveland, OH 44195, USA; ^2^Department of Internal Medicine, University of Cincinnati, 231 Albert Sabin Way, ML 0535, Cincinnati, OH 45267, USA; ^3^Department of Mathematics, Cleveland State University, 2121 Euclid Ave., Cleveland, OH 44115, USA

Cardiovascular disease (CVD) remains the leading cause of death globally. The identification of traditional risk factors such as age, hypercholesterolemia, hypertension, diabetes mellitus, and smoking has improved primary prevention of CVD. However, the overall mortality related to cardiovascular disease is still rising. Further scientific advances have led to the discovery of a broad range of novel biomarkers associated with cardiovascular risks, including B-type natriuretic peptide (BNP), N-terminal prohormone BNP (NT-proBNP), troponin, C-reactive protein (CRP), myeloperoxidase (MPO), lipoprotein-associated phospholipase A2, fibrinogen, TMAO, and cystatin C. Although these biomarkers have a prognostic value independent of the previous traditional risk factors, only a few have become important diagnostic tools in clinical practice. BNP and NT-proBNP have proven clinical utility in the diagnosis of heart failure and/or heart failure exacerbation. Troponin has been used as a cardiac biomarker for diagnosis and risk stratification of patients with suspected acute coronary syndrome (ACS). The blood levels of high sensitivity CRP (hs CRP) have been used to assess the risk of CVD, heart attack, and stroke. Circulating levels of MPO have been used to predict risks of coronary heart disease. However, so far, none of these biomarkers has significantly improved distinction between health and disease status. There remain tremendous challenges to scientists and clinicians in the discovery of novel biomarkers that may improve risk prediction of CVD, monitor disease progression, and potentially be used as therapeutic targets before clinical signs and symptoms appear.

This special issue contains nine papers, covering several aspects of biomarkers associated with cardiovascular disease. In six of the nine papers, the authors conduct small-scale retrospective cross-sectional or prospective clinical human studies to evaluate the clinical relevance of different biomarkers related to diverse disease conditions such as type 2 diabetes, acute ischemic stroke, heart failure, and aortic valve calcification. The biomarkers reported in this issue are miRNAs, cytokine IL-37, urinary NGAL, insulin resistance, metalloproteinase-8, metalloproteinase-1, parathyroid hormone, and lactate. Although respective authors have provided some evidence to show the promising prognostic value of these biomarkers, it is still unknown whether these markers will have a diagnostic impact or clinical implications. Also included in this special issue are three interesting review papers that discussed the effect of multiple vitamins, platelet miRNA expression profile, and ischemia-modified albumin on disease status.

In the paper “Plasma IL-37 Elevated in Patients with Chronic Heart Failure and Predicted Major Adverse Cardiac Events: A 1-Year Follow-Up Study,” X. Shou et al. provided evidence that circulating level of anti-inflammatory cytokine IL-37 is associated with increased risks for chronic heart failure.

In the paper “Parathyroid Hormone Levels in the Prediction of Ischemic Stroke Risk,” G. Çelik et al. performed a prospective study that examined parathyroid hormone (PTH) and/or 1,25-dihydroxyvitamin D (1, 25(OH)2 D) levels in 100 subjects who had acute ischemic stroke and 100 healthy control subjects. Their results suggested that PTH and 1,25(OH)2 D levels together may promote the determination of stroke risks.

In the paper “Handheld Capillary Blood Lactate Analyzer as an Accessible and Cost-Effective Prognostic Tool for the Assessment of Death and Heart Failure Occurrence during Long-Term Follow-Up,” G. M. Kubiak et al. measured capillary blood lactate levels with a handheld analyzer in a total of 145 consecutive patients admitted to hospitals after acute myocardial infarction. The authors demonstrated that lactate levels may serve as a potential prognostic marker of late onset heart failure and death after acute myocardial infarction.

In the paper “Is Urinary NGAL Determination Useful for Monitoring Kidney Function and Assessment of Cardiovascular Disease? A 12-Month Observation of Patients with type 2 Diabetes,” A Żyłka et al. assessed kidney function by monitoring the levels of urinary neutrophil gelatinase-associated lipocalin (uNGAL) in patients with type 2 diabetes mellitus (T2DM) after initiation of nephron-protective treatment in a one-year follow-up study. The authors found that better glycemic control in T2DM patients results in a significant decrease in uNGAL which is correlated with a significant improvement in renal function and the lowering of cardiovascular risk.

In the paper “Insulin Resistance in Adipose Tissue but Not in Liver Is Associated with Aortic Valve Calcification,” the authors present a cross-sectional clinical study with 1201 study population participating in the Genetics of Atherosclerotic Disease study. They discovered that adipose tissue insulin resistance is positively associated with the prevalent risks for aortic valve calcification (OR: 2.40: 95% CI: 1.30–4.43). They also found no association between adipose tissue insulin resistance and coronary artery calcification.

The paper by J. Mieczkowska et al. showed that circulating levels of inflammatory biomarker metalloproteinase-8 (MMP-8) and tissue inhibitor of metalloproteinase-1 (TIMP-1) are negatively correlated with a heart rate in postmenopausal women during exercise treadmill testing.

The review paper by I. Mozos et al. discussed the latest studies related to the effect of multiple vitamins on arterial stiffness. They suggested that vitamins A, B12, D, K, C, and E may be potential biomarkers for arterial stiffness.

The review by M. Bijak et al. touches upon a recently discovered diagnostic value of profiling miRNAs in platelets of diseased versus heathy controls. This review summarized interesting and thought-provoking studies implicating the role of platelet miRNAs in various diseases. More specifically, the described studies highlighted the role of (1) miRNA-340/miRNA-624 in premature coronary artery disease patients, (2) miRNA150 in heart failure with atrial fibrillation, (3) miRNA-154/miRNA-329/miRNA-376 in sickle cell disease, and (4) miRNA-144/miRNA-223/miRNA-146a in type 2 diabetes patients with ischemic stroke.

In their review, I. Oran et al. proposed a revised concept of fatty acid-occupied albumin, rather than ischemia-modified albumin, as a biomarker of acute coronary syndrome.

This special issue is aimed to broaden our knowledge on biomarkers of cardiovascular disease.

